# Do we really listen?

**DOI:** 10.1192/j.eurpsy.2021.981

**Published:** 2021-08-13

**Authors:** P. García Vázquez, R. Gomez Martinez

**Affiliations:** Psiquiatría, Complejo Asistencial Universitario León, León, Spain

**Keywords:** satisfaction, clinical evaluation, subjective, Day hospital

## Abstract

**Introduction:**

The improvement perceived by the patients is a subjective measure of the psychic state, while the clinical evaluation corresponds to an objective evaluation of the psychopathological improvement performed by a psychiatrist. It is therefore relevant to evaluate whether these parameters evolve in a common way after patients have undergone an intervention in Day Hospital focusing on first psychotic episodes.

**Objectives:**

Study the relationship between subjective improvement and clinical evaluation.

**Methods:**

This is a prospective study, which includes consecutive patients admitted to the Day Hospital during 2018. Their objective clinical improvement was assessed by means of the PANSS and GAF scales at admission and discharge. Subjective clinical improvement was assessed using an anonymous Likert scale with a score between 1 and 7. Sociodemographic data and other satisfaction parameters were also collected. A statistical analysis was performed using Pearson’s correlation.

**Results:**

A total of 73 patients were included. The perception of improvement on the part of the patients is very high presenting average values close to the maximum in almost all the evaluated items. The correlation between subjective improvement and PANSS variation presented a Pearson value 0.008; p = .957 and with the GAF variation presented a Pearson correlation of -0.066; p = .578 which indicates that there is no significant correlation between the variables.

**Table:** 

Diagnostic groups		
	Frequency	Percentage
Drugs	1	1,4
Psychosis	37	50.7
Affective	21	28.8
Neurosis	10	13.7
Personality	3	4.1
Total	72

**Conclusions:**

Clinical evaluation and subjective perception of improvement are independent parameters.

